# Acoustofluidic Chromatography for Extracellular Vesicle Enrichment from 4 μL Blood Plasma Samples

**DOI:** 10.1021/acs.analchem.4c06105

**Published:** 2025-03-13

**Authors:** Michael S. Gerlt, Thomas Laurell

**Affiliations:** Acoustofluidics Group, Lund University, Lund 221 00, Sweden

## Abstract

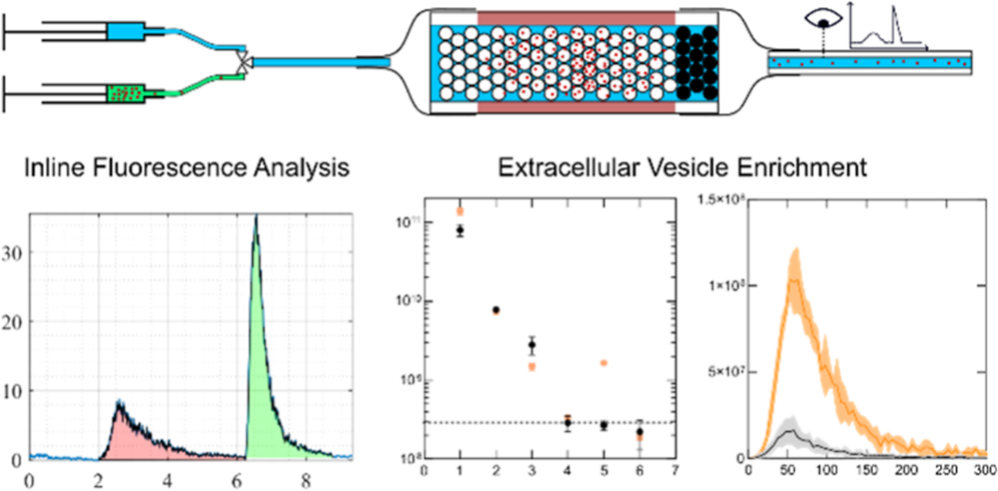

We present a novel acoustofluidic chromatography platform for high-throughput nanoparticle trapping and enrichment, with a focus on extracellular vesicles (EVs) from blood plasma. The system features a packed bed of polystyrene beads inside a rectangular glass capillary, acoustically actuated by a piezoelectric element. Using fluorescent polystyrene nanoparticles as small as 25 nm, we characterized device performance across a frequency range of 0.45–4 MHz, demonstrating particle trapping at all tested frequencies. The platform achieved recoveries of up to 42.9 ± 3.2% at input powers as low as 55 mW and operated at high flow rates of up to 200 μL/min. Trapping capacity reached 6.7 × 10^9^ ± 2.5 × 10^9^ particles for 25 nm polystyrene beads. For EV isolation, processing just 4 μL of blood plasma yielded 2 × 10^8^ washed EV-sized particles eluted in 100 μL within 8 min. Micro BCA analysis confirmed a plasma protein background below 2 μg/mL, enabling downstream mass spectrometry. This platform provides an efficient, high-throughput approach for nanoparticle trapping and EV enrichment with minimal sample volumes, offering potential applications in diagnostics and therapeutic development. Future work will focus on optimizing bead properties for EV subpopulation separation and scaling the system for clinical applications.

## Introduction

Extracellular vesicles (EVs) are membrane-bound nanoparticles secreted by nearly all mammalian cells. They play a crucial role in intercellular communication by transporting genetic and proteomic material. Due to their ability to reflect the physiological state of their cells of origin, EVs have emerged as promising biomarkers for early disease detection.^2^ Additionally, their natural material transport properties, long-term circulatory stability, and biocompatibility make them attractive candidates for drug delivery applications.^3^

Among the various biofluids studied for EV research, blood plasma is particularly relevant due to its diagnostic potential and accessibility.^4^ However, isolating EVs from plasma is challenging due to their small size (30–1000 nm) and the presence of high concentrations of lipoproteins and abundant plasma proteins.^5^ Traditional EV isolation methods,^6^ including ultracentrifugation,^7^ polymer precipitation,^8^ filtration,^9^ size-exclusion chromatography,^10^ and affinity precipitation,^11^ suffer from drawbacks such as long processing times, large sample volume requirements, high costs, and inconsistent purity and recovery.^12^ These limitations have driven the development of alternative isolation methods, particularly microfluidic-based approaches that enable processing of smaller sample volumes with higher purity.^13^

Microfluidic EV isolation methods can be broadly categorized into passive and active techniques. Passive methods exploit physical particle properties such as size, shape, or density. They are based on mechanical filters (centrifugal disc microfluidics,^14^ ciliated micropillars,^15^ Exodus^16^), hydrodynamic forces (pinched flow fractionation,^17^ asymmetric field flow fractionation,^18^ deterministic lateral displacement,^19^ viscoelastic separation^20^), or functionalized surfaces.^21,22^ Active methods, on the other hand, employ external forces such as magnetic,^23,24^ electric,^25,26^ thermal,^27^ or acoustic^28−30^ fields to manipulate EVs. A summary of the advantages and limitations of microfluidic EV isolation methods is provided in Table 1.

**Table 1:** Advantages and Limitations of Microfluidic Methods for EV Isolation

method	advantages	limitations
mechanical filters,^14−16^	- applicable for point of care	- shear-induced damage
	- automated	- clogging
hydrodynamic forces,^17−20^	- high throughput	- sample dilution
	- simple	- clogging
functionalized surfaces^21,22^	- highly specific	- costly reagents
	- easily scalable	- release challenging
magnetophoresis,^23,24^	- highly specific	- costly reagents
	- easily scalable	- risk of aggregation
electrophoresis^25,26^	- label-free	- low throughput
	- high resolution separation of charged EV subtypes	- contamination with other charged molecules
thermophoresis^27^	- gentle and label-free	- limited throughput and scalability
	- EV subtypes based on size, composition, hydration shell	- sensitive to buffer conditions
acoustophoresis^28−30^	- gentle and label-free	- requires precise acoustic control
	- continuous separation	- potential loss of smaller EVs (both drawbacks are alleviated in this manuscript)

This study focuses on acoustic particle manipulation due to its label-free and gentle nature, as well as its ability to target nanoscale particles. Acoustic methods are categorized into surface acoustic wave (SAW) and bulk acoustic wave (BAW) approaches. Surface acoustic wave -based devices, operating at high MHz to GHz frequencies, allow for precise particle separation, including isolation of exomeres (30–50 nm),^30,33,34^ and can be used to trap EVs by exciting their resonance frequency.^28^ However, SAW devices require costly microfabrication in cleanroom environments and typically generate lower acoustic energy densities, limiting throughput.

Bulk acoustic wave devices, in contrast, utilize piezoelectric elements attached to microfluidic channels—typically made of silicon or glass—to induce ultrasound at lower frequencies (MHz range). While direct manipulation of EVs is not feasible at these frequencies, EV trapping can be achieved through secondary acoustic forces generated by “seed particles” that scatter acoustic waves.^35^ Pioneering work on acoustofluidic EV isolation by Evander et al.^36,37^ demonstrated proof-of-concept applications, with subsequent improvements in throughput and trapping capacity.^38,39^ However, current designs are limited by their reliance on precise frequency-tracking algorithms, sensitivity to temperature fluctuations, and restricted trapping capacity.

In this study, we introduce acoustofluidic chromatography—a novel approach designed to overcome these limitations by enabling high-throughput and high-capacity trapping of nanoscale particles with minimal sample volumes. Analogous to traditional chromatography, our method employs micrometre-sized “seed particles” packed within a glass capillary. Upon acoustic excitation, these particles generate forces that attract and retain smaller nanoparticles such as EVs. Unlike previous designs,^28,29,40,50^ our system operates across a wide frequency range without requiring precise resonance matching of the beads or device.

We developed a setup consisting of syringe pumps and a fluorescence microscope, allowing characterization of key performance metrics such as recovery, flow rate, and adsorption efficiency. Our results demonstrate particle trapping across a broad frequency range (0.45–4 MHz), high flow rates (up to 100 μL/min), and recoveries of up to 42.9 ± 3.2%. Furthermore, we successfully enriched EVs from as little as 4 μL of human blood plasma, isolating up to 2 × 10^8^ EV-sized particles eluted in 100 μL within just 8 min, with minimal protein contamination (<2 μg/mL, confirmed by micro BCA analysis).

These findings highlight the potential of acoustofluidic chromatography for scalable, high-purity EV isolation, with promising applications in diagnostics and therapeutic development.

## Materials and Methods

### Device Fabrication

The trapping device was constructed using a rectangular glass capillary (20 mm length, 4 mm width, 0.4 mm height, 0.28 mm wall thickness, 2540 – Rectangle Glass Tubing, VitroCom, USA). A piezoelectric element (10 mm length, 4 mm width, 0.5 mm thickness, PZ26, CTS, Denmark) was attached to the bottom of the capillary using conductive epoxy (H20E, EPO-TEK). Copper wires (0.15 mm diameter) were glued to the piezoelectric element using conductive silver paste, and mechanical stability was enhanced by securing them with instant glue. To form the packed bed, the capillary was partially filled with polystyrene beads (100 μm diameter, 10% w/v, PS-R-100.0, Microparticles GmbH). The bottom 4 mm of the capillary was filled first, with beads retained by a filter paper to prevent their exit. The beads were then heated to their glass transition temperature (∼120 °C) using a heat gun, causing them to fuse and form a stable frit. Subsequently, the capillary was completely filled with additional polystyrene beads, with the filling process conducted in an ultrasound bath while perfusing the capillary to ensure uniform packing in a hexagonal arrangement typical of lattice packed beads (Figure S1). After filling the trapping device, we did not sinter particles close to the inlet to enable more efficient cleaning and reloading of fresh seed particles (see Experimental procedure).

**Figure 1:**
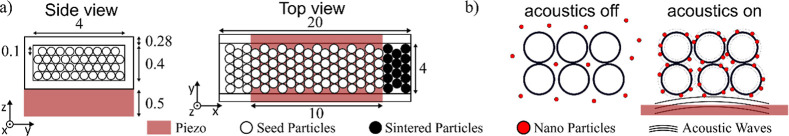
Acoustofluidic chromatography device. (a) Schematic of the trapping device. All units are given in mm. (b) Schematic of the trapping mechanism. Acoustic waves from the piezoelectric element are scattered by the seed particles, which leads to secondary acoustic interaction forces with the nanoparticles resulting in strong attraction to the seed particles.

Fluidic connections were made by attaching silicone tubing (0.8 mm inner diameter, 3 mm outer diameter, VWR, Sweden) to the capillary ends using heat-shrink tubing. A schematic of the trapping device cross sections can be seen in Figure 1a.

### Working Principle

Upon activation, the piezoelectric element generates acoustic waves, which are scattered by the seed particles within the glass capillary. These scattered waves interact with nanoparticles, such as EVs, flowing through the device, exerting secondary acoustic radiation forces that drive them toward the seed particles (Figure 1b). Since the strength of these secondary forces rapidly decreases with distance,^41^ additional mechanisms likely contribute to nanoparticle trapping. These include acoustic streaming and electrostatic interactions, which promote attraction to the seed particles, as well as hydrodynamic shielding and hydrophobic/hydrophilic interactions, which aid in particle retention within the device.

While our current setup does not allow for direct quantification of the relative contributions of these trapping mechanisms, we observe a clear dependence of particle recovery and adsorption on flow rate. These observations provide indirect insights into the factors influencing the trapping process, suggesting a complex interplay between acoustic, hydrodynamic, and surface interactions.

### Experimental Setup

**Figure 2:**
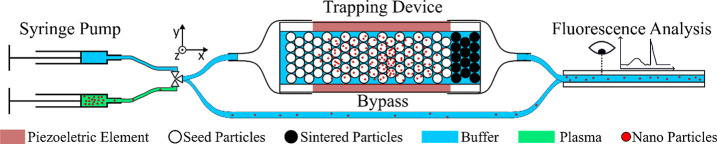
Experimental Setup for real time analysis of recovery in flow. Schematic of the experimental setup consisting of two syringe pumps, the trapping device and a fluorescent microscope. The syringe pumps are connected to a valve for automated switching. The microscope camera records the fluorescent intensity. Before every set of experiments, particles are flown through the bypass to measure the fluorescent signal of the inserted particles.

The experimental setup consisted of two syringe pumps (Nemesys S, Cetoni, Germany), each equipped with a syringe (Hamilton, Switzerland). One syringe contained the sample (1 mL), and the other held clean phosphate-buffered saline (PBS, 5 mL). The pumps were connected to a valve system, which was conveniently sitting on the syringe pump modules and controlled by the same software, for automated switching between the sample and buffer solutions. Teflon tubing (1.58 mm outer diameter, 0.3 mm inner diameter, 58698-U, Supelco, USA) was used for all fluidic connections. The inlet of the trapping device was connected to the valve and the outlet to a square glass capillary (analysis capillary) (0.4 mm inner diameter, 0.2 mm wall thickness, 8240 – Square Glass Tubing, VitroCom, USA), which was monitored by a microscope (SZX16, Olympus, Japan). The microscope was equipped with a blue LED (450 nm wavelength, pE-300 white, CoolLED, UK), a GFP filter set (excitation 457–487 nm, emission 502–538 nm, Edmund Optics, UK), and a camera (U3–3880CP-M-GL, IDS, Germany) for fluorescent signal detection. To obtain a fluorescence graph for the input particles, the trapping device was replaced by tubing with the same length and the particles were flown directly into the square capillary for analysis (Bypass). The overall setup is shown in Figure 2.

The piezoelectric element was driven by a function generator (AFG3022B, Tektronix, Germany) with the signal amplified using a power amplifier (150A100D, AMETEK, USA). Voltage and current across the piezoelectric element were monitored by an oscilloscope (TBS 2074b, Tektronix, Germany), and current measured via a current probe (CT2, Tektronix, Germany). The power was calculated by the oscilloscope by calculating the mean value of the voltage and current product. To manage temperature at elevated driving power, the piezoelectric element was cooled by a fan placed directly above the trapping device. Temperatures were continuously monitored with a PT1000 sensor affixed to the capillary and a thermocouple connected to a temperature recorder (88378 AZ-Y2022, AZ-Instruments, Taiwan). The temperature readings were verified to ensure consistency. A picture of the experimental setup can be seen in the Supporting Information (Figure S-2).

### Sample Preparation

For device characterization, fluorescent polystyrene beads (1.9 μm diameter, G0200; 0.27 μm diameter, G300, 1% solid, excitation/emission 468/508 nm, ThermoFisher, USA, and 0.1 μm diameter, 29-00-102; 0.025 μm diameter, 29-00-251, 10 mg/mL, excitation/emission 475/510 nm, Micromod, Germany) were diluted in PBS with 0.4% added bovine serum albumin (BSA). The BSA reduced nonspecific binding and aggregation. Dilutions ranged from 1:10 to 1:100000, depending on the desired particle concentration for each experiment. For extracellular vesicle (EV) capture experiments, whole blood samples were collected from anonymized healthy volunteers providing signed informed consent at the Biomedical Center, Lund University (Lund, Sweden) according to a protocol approved by the Swedish ethical review authority (ref. no. 2020-05818). The blood underwent triple centrifugation following a protocol specifically designed for EVs reported in the literature^42,43^ resulting in platelet-poor plasma. The plasma was verified by flow cytometry to contain fewer than 10 platelets/mL and was then used for the EV isolation experiments.

### Experimental Procedure

**Figure 3:**
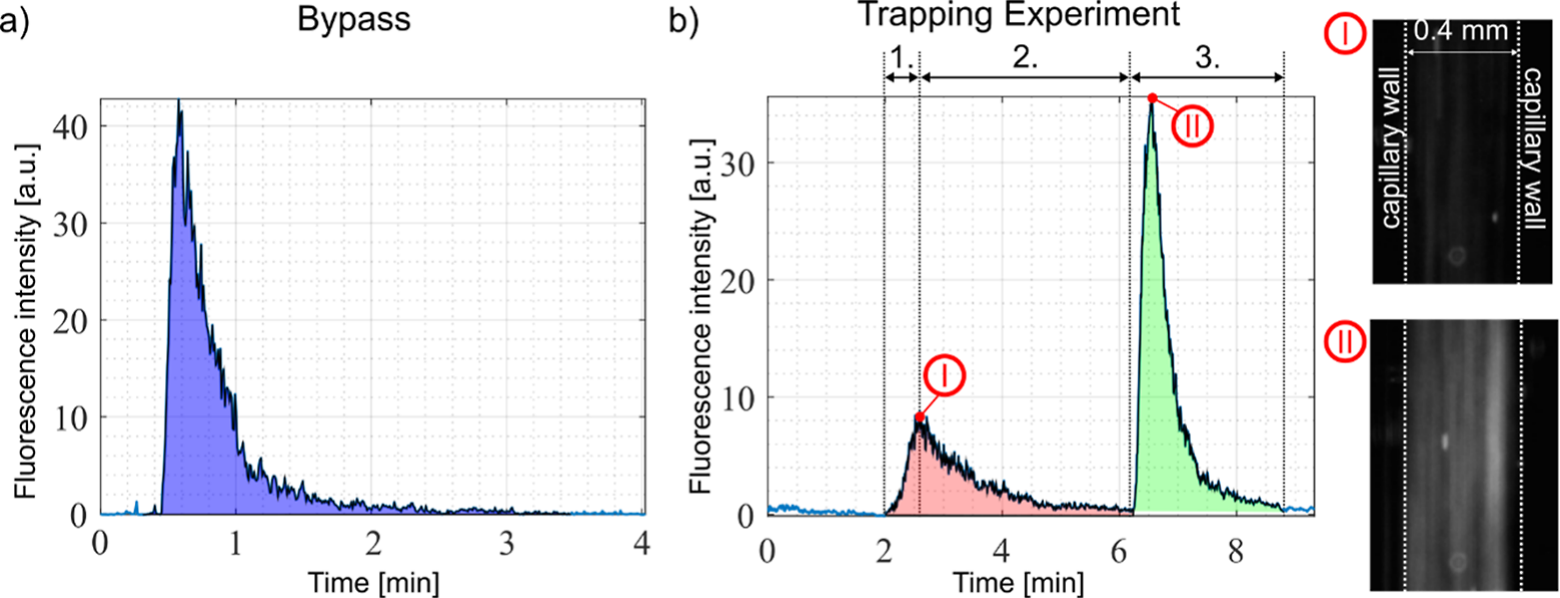
Experimental analysis. (a) Fluorescence intensity graph attained by flowing 10 μL of 2 μm diameter polystyrene beads at a concentration of 2.4 × 10^7^ particles/mL through a tube connected to the analysis capillary (bypass). (b) Fluorescence intensity graph attained from an experiment using the same number of particles as in (a) following the experimental procedure: 1. insert sample, 2. wash with PBS, 3. turn off the ultrasound and release trapped particles. The red area corresponds to beads that passed the packed bed even though the ultrasound was active (lost particles). The green area corresponds to beads that were released when the ultrasound was turned off (recovered particles). Right side: microscope images of the analysis capillary during a typical experiment clearly demonstrating the difference in fluorescent intensity during the different steps.

Before each experiment, the trapping device was purged by sonicating the packed bed in an ultrasound bath while partly pulling out the seed particles and flushing them back in releasing all adsorbed particles from previous sample runs. If contamination was detected under a fluorescent microscope, the packed bed was exchanged completely. For every new number of particles (combination of concentration and input volume) the trapping device was disconnected and bypassed using standard tubing to attain a fluorescence elution peak representing the input particles (Figure 3a). In the beginning of each trapping experiment, the piezoelectric element was turned on for at least 1 min to equilibrate the system. The following protocol was used for experiments with fluorescent beads:

1. The trapping device was flushed with PBS and the fluorescent signal was recorded for 30 s to establish a baseline.
2. The valve was then switched to the sample syringe, and a controlled volume of sample (10–100 μL) was injected into the system (1. In Figure 3b).
3. After sample injection, the valve was switched to buffer, and a washing step was performed (2. In Figure 3b).
4. The ultrasound was turned off to release and elute the trapped particles, generating a fluorescence elution peak. Recording continued until the signal returned to baseline (3. In Figure 3b).

For EV capture experiments, human blood plasma samples were diluted 1:10 with PBS, and 40 μL of diluted plasma (equivalent to 4 μL of pure plasma) was inserted during the sample injection step. The washing step used 360 μL of buffer and the flow rate of the whole experiment was chosen to be 50 μL/min. Preliminary tests showed no increase in particle concentration under the standard protocol, so a modification in the release step 4 was introduced: after switching off the ultrasound, the flow rate was abruptly increased to 200 μL/min to assist in releasing the captured particles.

### Experimental Analysis

During each experiment, the fluorescence signal of particles flowing through the analysis capillary was recorded and analyzed using a custom MATLAB script, calculating the average fluorescence intensity for each image and integrating the area under the curve. The camera and excitation settings were chosen such that no pixel was saturated. For each new number of particles, we first bypassed the trapping device to obtain a representative fluorescent signal of all inserted particles (Figure 3a, blue area). In the trapping experiments, we can distinguish between beads passing through the packed bed during trapping (flow through fraction, red area) and those released after ultrasound deactivation (recovered fraction, green area) (Figure 3b). For device characterization, we calculated the following values based on the fluorescence plot we obtained
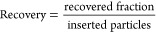
1
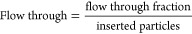
2
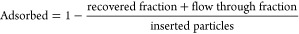
3

**Figure 4:**
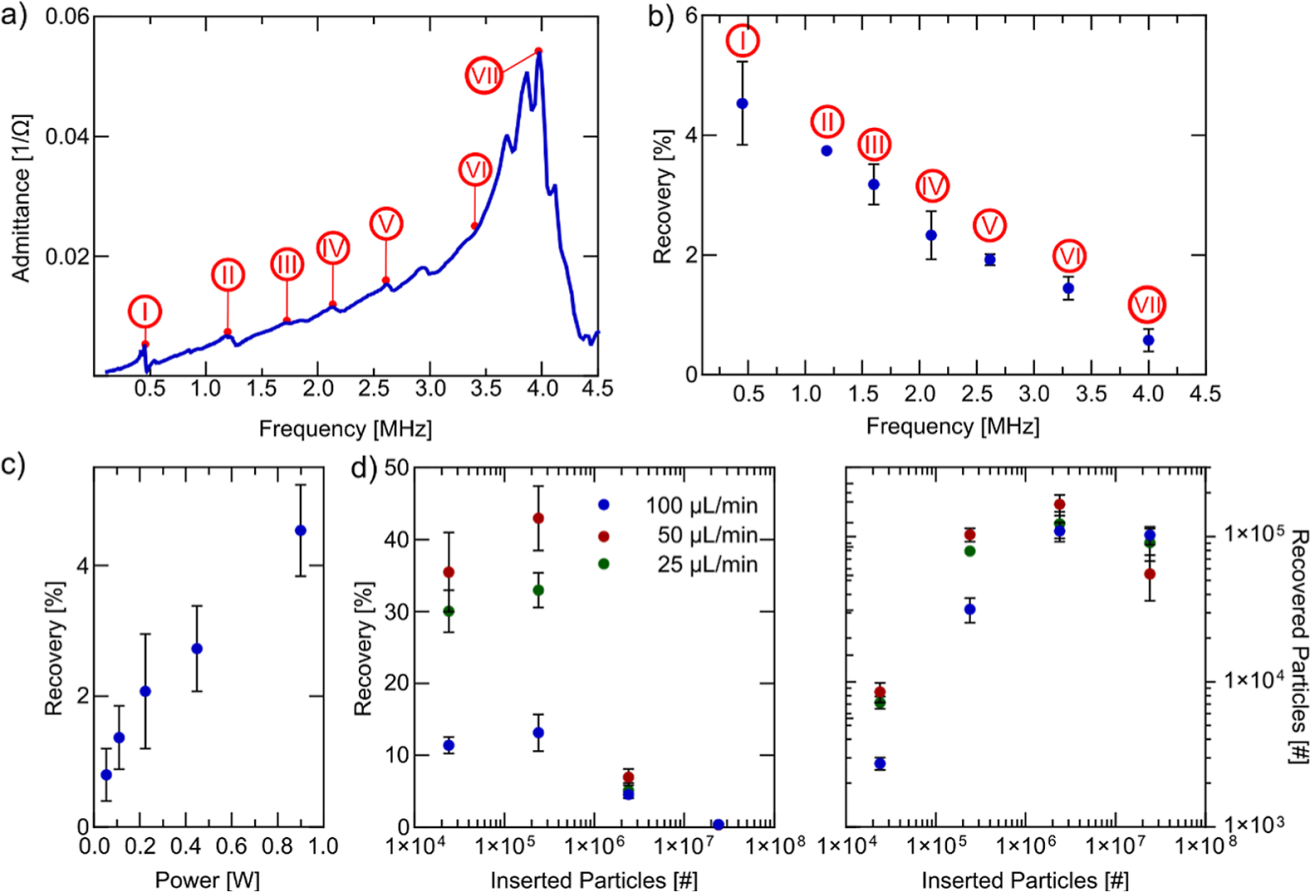
Device characterization. (a) Impedance spectrum of the trapping device showcasing various resonances. (b) Trapping experiments performed at specific frequencies using the experimental procedure with 1.9 μm fluorescent PS particles diluted 1:100 with PBS. The flow rate was 100 μL/min and the power supplied to the piezoelectric element 900 mW. (c) Influence of the input power on the recovery. Decreasing the input power leads to lower recovery. (d) Influence of the inserted number of particles and flow rate on the recovery. Trapping capacity can be estimated to be around 1 × 10^5^ Particles. All error bars were generated by three independent experiments.

For EV experiments, fluorescence analysis was not applicable since EVs are not intrinsically fluorescent. Instead, 100 μL aliquots were collected in 0.5 mL Eppendorf tubes (Protein LoBind Tubes, Eppendorf, Germany) consecutively and analyzed using nanoparticle tracking analysis (NTA, NanoSight Pro, Malvern Panalytical, UK). The samples were perfused through the NTA at a flow rate of 10 μL/min, and statistically significant data required the collection of at least 10000 valid tracks. The detection limit for the NTA was approximately 2 × 10^8^ particles/mL. Total protein concentration in the samples was measured using a micro-BCA assay (micro-BCA Protein Assay Kit, ThermoFisher, USA), following the manufacturer’s instructions. A standard curve ranging from 2.5 to 200 μg/mL was used. Briefly, 150 μL of working reagent was mixed with 150 μL of diluted sample, incubated at 37 °C for 2 h, and absorbance was measured at 562 nm using a plate reader (Infinite F Nano+, Tecan, Switzerland). Protein concentration was then calculated from the standard curve. The admittance spectrum (Figure 4a) was generated using an impedance analyzer (IM7581, Hioki, Japan).

## Results and Discussion

### Device Characterization

We first conducted a comprehensive characterization of the trapping device. The impedance spectrum of the piezoelectric element attached to the trapping device (Figure 4a) revealed multiple resonances, with the resonances at 0.45 MHz (I) and 4 MHz (VII) corresponding to the width and thickness resonances of the piezoelectric element, respectively.

Next, we conducted trapping experiments following the experimental procedure (as specified in the Materials and Methods section) using 1.9 μm fluorescent polystyrene (PS) particles (1% v/v) suspended in PBS at a 1:100 dilution. We inserted 100 μL of the particle suspension leading to a total of 2.38 × 10^6^ particles inserted following by a PBS wash with 150 μL. We maintained a flow rate of 100 μL/min and supplied a constant power of 900 mW to the piezoelectric element. To assess frequency dependence, we varied the frequency from 0.45 to 4 MHz (Figure 4b). The recovery was highest at the width resonance (0.45 MHz) and decreased progressively with higher frequencies. Interestingly, particle trapping occurred across the entire frequency range, independent of resonance, albeit with varying recoveries. Data including all performance metrics can be found in Table S1. It should be noted that at 900 mW, the voltage required for the width resonance (0.45 MHz) was significantly higher requiring 50 V_pp_ compared to the thickness resonance (4 MHz) requiring 7 V_pp_, due to the lower admittance at the width resonance. Therefore, while trapping at the thickness resonance might be more practical for systems where voltage amplification is undesirable, the higher recovery at 0.45 MHz justifies its use for subsequent experiments.

We then varied the input power to assess its impact on particle recovery. As expected, lower input power resulted in decreased recovery; however, even at the lowest tested power (55 mW), particle trapping remained detectable (Figure 4c). A comprehensive summary of performance metrics is provided in Table S2. For subsequent experiments, we set the input power to 900 mW, as temperatures at the piezoelectric element (35 °C) and the bottom of the capillary (30 °C) approached safe operational limits for biological samples while maintaining high recovery efficiency.

Next, we investigated the effects of particle concentration and flow rate. At low particle concentrations (<2.38 × 10^5^), reducing the flow rate from 100 to 50 μL/min significantly improved particle recovery (Figure 4d). However, further reducing the flow rate to 25 μL/min led to a decline in recovery despite a reduction in the flow-through fraction. This suggests increased particle adsorption within the packed bed, potentially due to enhanced hydrophobic interactions (Table S3). Consequently, 50 μL/min emerged as the optimal flow rate and has been used in all further experiments with PS particles.

Increasing the number of inserted particles revealed a saturation effect, indicating a capacity limit of approximately 2 × 10^5^ particles for 1.9 μm diameter polystyrene beads. Notably, the elution volume remained consistently around 20 μL, regardless of flow rate or particle concentration, highlighting the device’s ability to effectively concentrate particles within a minimal fluid volume (Figure S3). Additionally, the entire trapping process was completed in just 3 min at 100 μL/min and 8 min at the lowest tested flow rate of 25 μL/min. This flexibility allows for significant reductions in experimental time depending on recovery requirements.

In summary, we identified the optimal operating conditions for the trapping device as 450 kHz (width resonance), 900 mW input power, and a flow rate of 50 μL/min when working with low particle concentrations to maximize recovery efficiency.

### Nanometer-Particle Trapping

Following the characterization with 1.9 μm PS particles, we introduced nanoparticles into our system to assess the influence of particle size on performance metrics.

**Figure 5:**
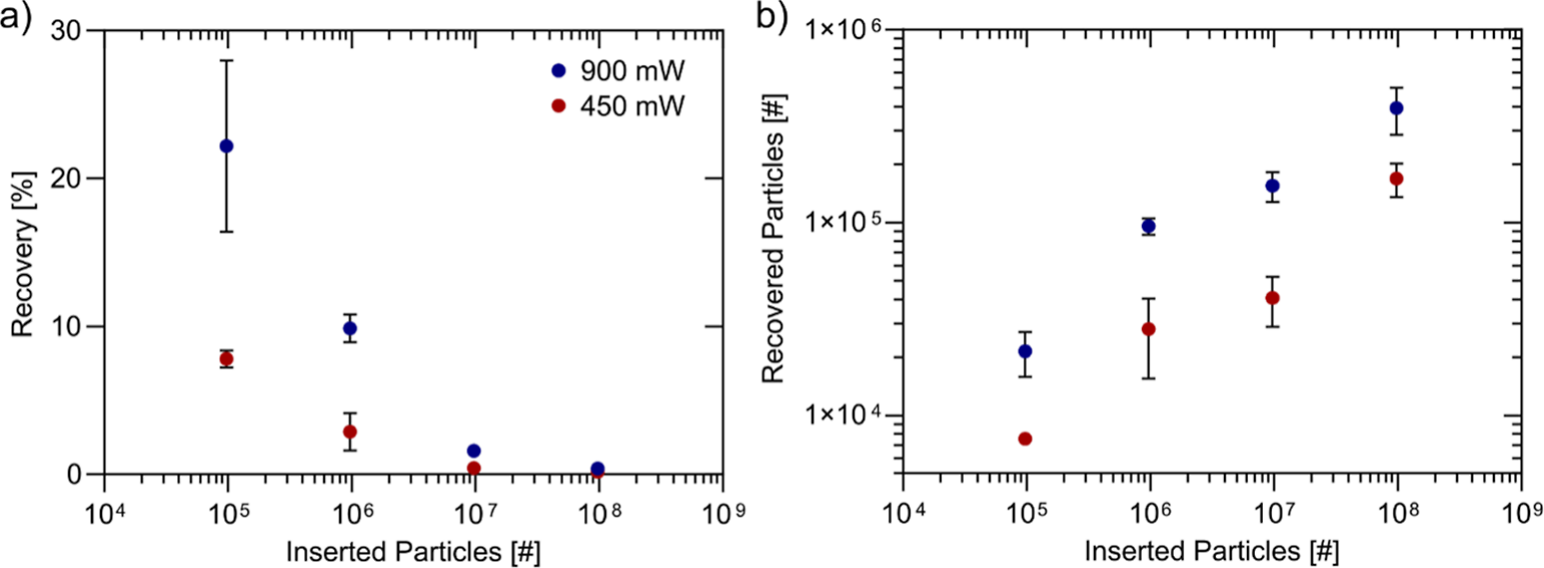
270 nm-particle trapping. (a) Influence of inserted particle number and applied power on recovery. Lower inserted particle number and higher power lead to higher recoveries. (b) The estimated trapping capacity for 270 nm particles is ∼4 × 10^5^ Particles.

In the first set of experiments, we tested 270 nm diameter PS particles. Similar to larger particles, lower particle number and higher input power resulted in higher recovery (Figure 5a). Notably, when 9.7 × 10^4^ particles were introduced, the recovery for 270 nm particles was only 20% lower than that observed for 1.9 μm particles under similar conditions (Table 2). This finding is particularly interesting, as the acoustic attraction force, scaling with particle volume, should be significantly weaker for smaller particles. We hypothesize that hydrodynamic shielding may enhance recovery for these nanoscale particles, a concept we explore further in the next section. The estimated trapping capacity for 270 nm particles, determined by multiplying recovery by the number of inserted particles, was approximately 4 × 10^5^ particles (Figure 5b). Additional performance metrics for 450 mW are provided in Table S4.

**Table 2:** Performance Metrics of a Trapping Experiment Using 270 nm PS Particles Corresponding to Figure 5^a^

inserted particles [#]	9.7 × 10^4^	9.7 × 10^5^	9.7 × 10^6^	9.7 × 10^7^
recovery [%]	22.2 ± 4.7	9.9 ± 0.8	1.6 ± 0.2	0.4 ± 0.1
flow through [%]	55.5 ± 3.7	51.4 ± 6.9	58.1 ± 4.6	57.6 ± 0.6
adsorbed [%]	22.4 ± 6.7	38.7 ± 6.2	40.3 ± 4.7	42.0 ± 0.5
recovered particles [#]	2.7 × 10^4^ ± 4.4 × 10^3^	1.6 × 10^5^ ± 2.7 × 10^4^	2.6 × 10^5^ ± 3.3 × 10^4^	3.9 × 10^5^ ± 8.8 × 10^4^

a Inserted particles: 270 nm PS. Power: 900 mW. Flow rate: 50 μL/min.

**Figure 6:**
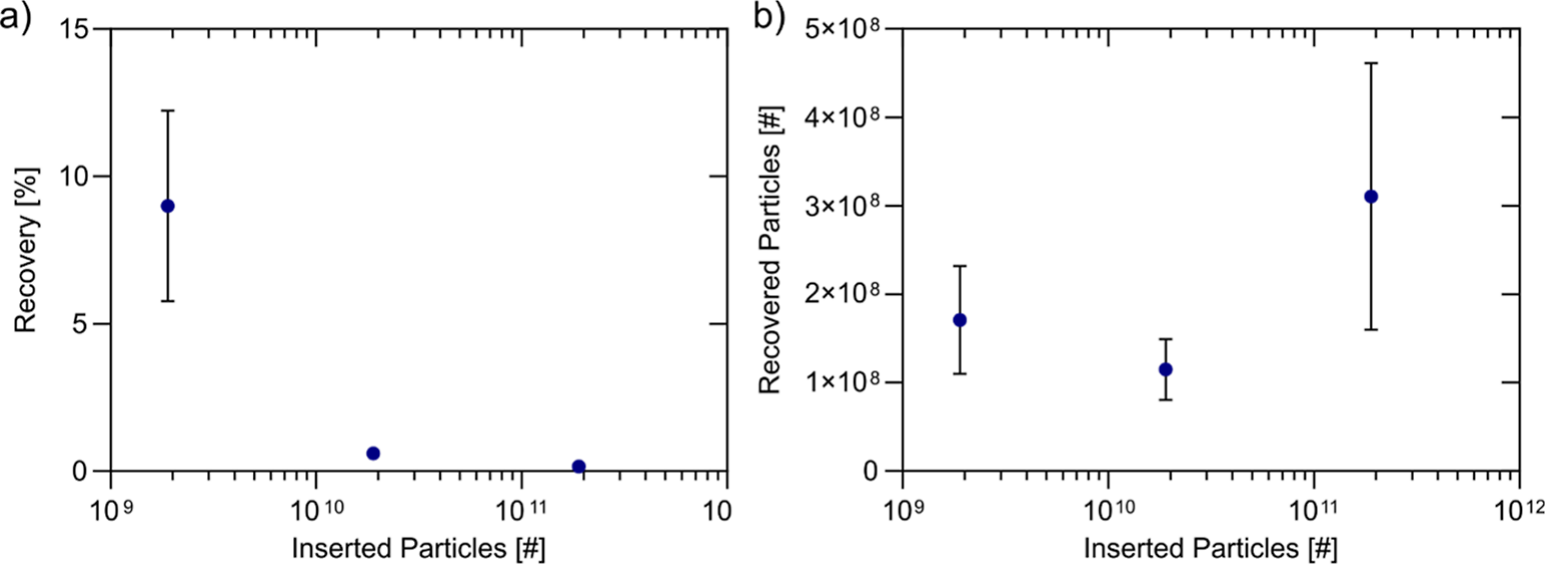
100 nm-particle trapping. (a) Influence of inserted particle number and applied power on recovery. (b) The capacity of the system for 100 nm PS particles lies around 2 × 10^8^ Particles.

To more closely mimic the size of extracellular vesicles, we further reduced the particle diameter to 100 nm. Due to the decreasing fluorescence signal with smaller particle sizes, we increased the inserted particle number to at least 1.9 × 10^9^. Remarkably, even at these high particle concentrations, we achieved a recovery of 9.0 ± 2.6%, which is significantly higher than that reported for other seed particle based acoustic EV trapping devices.^44^ This improvement is likely due to a lower degree of particle adsorption within the packed bed compared to the 2 μm particles (Table 3). As the particle concentration increased, recovery decreased, allowing us to estimate the trapping capacity for 100 nm particles at approximately 2 × 10^8^ (Figure 6).

**Table 3:** Performance Metrics of a Trapping Experiment Using 100 nm PS Particles Correspponding to Figure 6^a^

inserted particles [#]	1.9 × 10^9^	1.9 × 10^10^	1.9 × 10^11^
recovery [%]	9.0 ± 2.6	0.6 ± 0.1	0.2 ± 0.1
flow through [%]	86.3 ± 4.2	86.1 ± 13.4	64.0 ± 1.4
adsorbed [%]	4.8 ± 4.8	13.3 ± 13.5	35.8 ± 1.4
recovered particles	1.7 × 10^8^ ± 5.0 × 10^7^	1.1 × 10^8^ ± 2.8 × 10^7^	3.1 × 10^8^ ± 1.2 × 10^8^

a Inserted particles: 100 nm PS. Power: 900 mW. Flow rate: 50 μL/min.

Finally, we tested 25 nm PS particles, a size below the detection limit of state-of-the-art NTA systems. We successfully recovered ∼6% of the inserted particles, suggesting a trapping capacity of approximately 7 × 10^9^ for 25 nm PS particles (Table 4).

**Table 4:** Performance Metrics of a Trapping Experiment Using 25 nm PS Particles^a^

	recovery [%]	flow through [%]	adsorbed [%]	recovered particles [#]
25 nm	5.6 ± 2.1	81.6 ± 1.4	12.9 ± 2.5	6.7 × 10^9^ ± 2.5 × 10^9^

a Inserted Particles: 1.2 × 10^11^ 25 nm PS. Power: 900 mW. Flow rate: 50 μL/min.

In conclusion, our findings demonstrate that particles as small as 25 nm can be effectively trapped in our device. Notably, the experiments were completed within just 6 min, making this method significantly faster than most nanoparticle enrichment techniques described in the literature. Furthermore, our results highlight a striking increase in trapping capacity with decreasing particle size, underscoring the scalability and efficiency of the device for nanoscale particle enrichment.

### Extracellular Vesicle Trapping from 4 μL of Blood Plasma

Following the successful results with the nanometer-scale polystyrene particles, we proceeded to experiments using blood plasma. Details on the experimental protocol and sample handling are provided in the Materials and Methods section. For extracellular vesicle (EV) trapping, we applied a frequency of 450 kHz to the piezoelectric element at a power of 900 mW, with a flow rate of 50 μL/min. Aliquots of 100 μL were sequentially collected and analyzed using nanoparticle tracking analysis (NTA) and micro-BCA (μBCA) assays.

**Figure 7:**
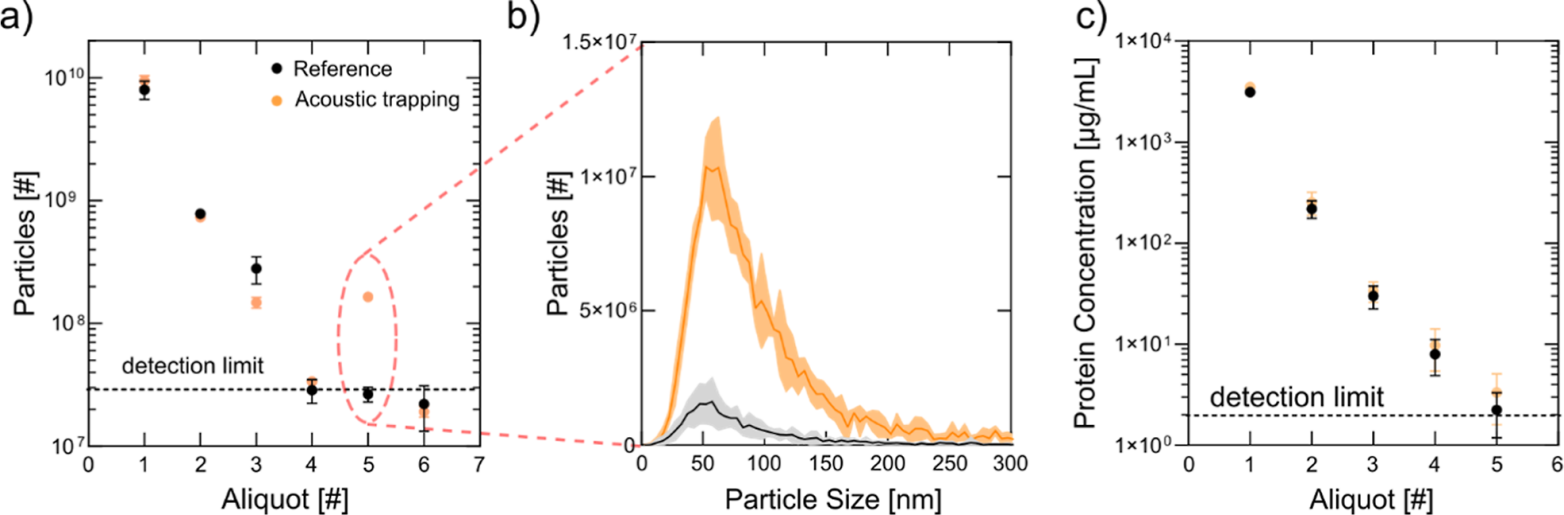
EVs can be efficiently trapped, washed and released on demand. (a) NTA measurement of the particle number in the collected 100 μL aliquots. The number of particles decreases with increasing washing volume i.e. aliquot number. When the acoustics is turned on in the beginning of the experiment (yellow dots) and turned off after 360 μL washing, a significant increase in particle number can be observed in the fifth aliquot compared to experiments without the use of ultrasound (black dots). All experiments were performed in triplicates represented by the error bars. Especially for the acoustic trapping, the error was too low to be displayed. (b) NTA measurement of the particle size vs particle number in aliquot 5. Particles mostly contributing are in the size range from 30 to 150 nm, typical for small EVs. (c) Micro-BCA measurement for protein concentration. The same trend as for the particle number can be seen–decreasing protein concentration with increasing washing, i.e. aliquot number. However, the protein concentration does not increase when EVs are released in aliquot 5 for the acoustic experiment indicating the efficiency of our washing protocol.

First, we flushed the plasma through the trapping device without activating the ultrasound to establish a reference elution profile. NTA results showed a steady decrease in particle number as the washing progressed. The particle number in the first aliquot was 8.0 ± 1.4 × 10^9^ particles, but after washing with 260 μL of PBS (fourth aliquot), the particle number dropped to the detection limit of the NTA (Figure 7a). Subsequent aliquots contained statistically insignificant particle numbers due to low track counts. Protein quantification using micro-BCA confirmed these findings, with the initial plasma protein concentration of 3122 ± 277 μg/mL significantly reduced to 2.25 ± 0.7 μg/mL after the fourth aliquot (Figure 7c). Further washing decreased protein levels below the detection limit of the plate reader. Based on these results, in the subsequent acoustic trapping studies we released trapped particles after washing with 360 μL of PBS (fifth aliquot). When ultrasound was applied at the start of the experiment and deactivated after the washing step, we observed a marked increase in number of particles in the fifth aliquot, with 1.6 ± 0.9 × 10^8^ particles released in the 100 μL aliquot. In contrast, the reference sample without acoustic trapping was below the detection limit (Figure 7a). Analyzing the size distribution of the released particles in the fifth aliquot revealed that the increase in number of particles was primarily due to particles in the EV size range (Figure 7b). Most of the particles contributing to the increase in recovered particle count were in the 50–60 nm range, consistent with the size of small EVs and lipoproteins, which are more abundant than larger EVs (200–1000 nm) .^6^ Notably, other isolation methods, including size exclusion chromatography and polymer precipitation, typically yield the highest particle concentrations in the 100–200 nm range.^45–47^

A key advantage of our approach is the high reproducibility of the recovered particle count, which contrasts with commercial seed particle-based acoustic EV trapping, where recovery can vary by an order of magnitude.^47^ Furthermore, μBCA assay results confirmed that protein concentration did not significantly increase after particle release, indicating that our washing protocol effectively removed plasma proteins (Figure 7c).

To assess recovery, we quantified input particle numbers in plasma using NTA, yielding approximately 1.2 × 10^10^ ± 8.7 × 10^8^ particles in a 4 μL volume. The recovered particle count aligned well with previously determined trapping capacities, with an estimated recovery of ∼1% (Table 5). As noted in comparative studies of isolation methods, acoustofluidic isolation prioritizes rapid processing times, small sample volumes, and high purity over absolute recovery efficiency.^46,47^

**Table 5:** Additional Analysis of the Acoustic Trapping Experiment Presented in Figure 7^a^

	recovery [ %]	flow through [ %]	adsorbed [ %]	recovered particles [#]
plasma	1.3 ± 0.1	70.4 ± 8.4	28.3 ± 3.7	1.7 × 10^8^ ± 1.0 × 10^6^

a Based on NTA measurements, the input particle number was 1.2 × 10^10^ ± 8.7 × 10^8^.

It is important to note that NTA-based recovery estimates have limitations. Since NTA cannot detect particles below 50 nm, the actual recovery is likely higher, given that our device efficiently traps and releases 25 nm particles. Additionally, NTA cannot distinguish between EVs and lipoproteins, which are far more abundant in blood plasma.^43^ The initial particle concentration of 3.1 × 10^12^ particles/mL strongly suggests a high lipoprotein content, as the typical EV concentration in plasma is in the range of ∼10^10^ EVs/mL.^48^ However, Lipoprotein concentration is likely to be much reduced in the recovered fraction due to repulsive acoustic forces on these particles,^49^ potentially enhancing EV recovery. Future experiments will focus on specific EV markers, potentially using nano flow cytometry, to eliminate the influence of lipoproteins on the results.

Finally, we observed that approximately 28% of the particles remained trapped in the device. Optimizing release strategies, such as surface modifications or external force application, could significantly improve recovery.

In summary, our system effectively traps and washes nanoparticles and successfully operates with biological fluids such as blood plasma, demonstrating its potential for EV isolation in bioanalytical applications.

**Figure 8:**
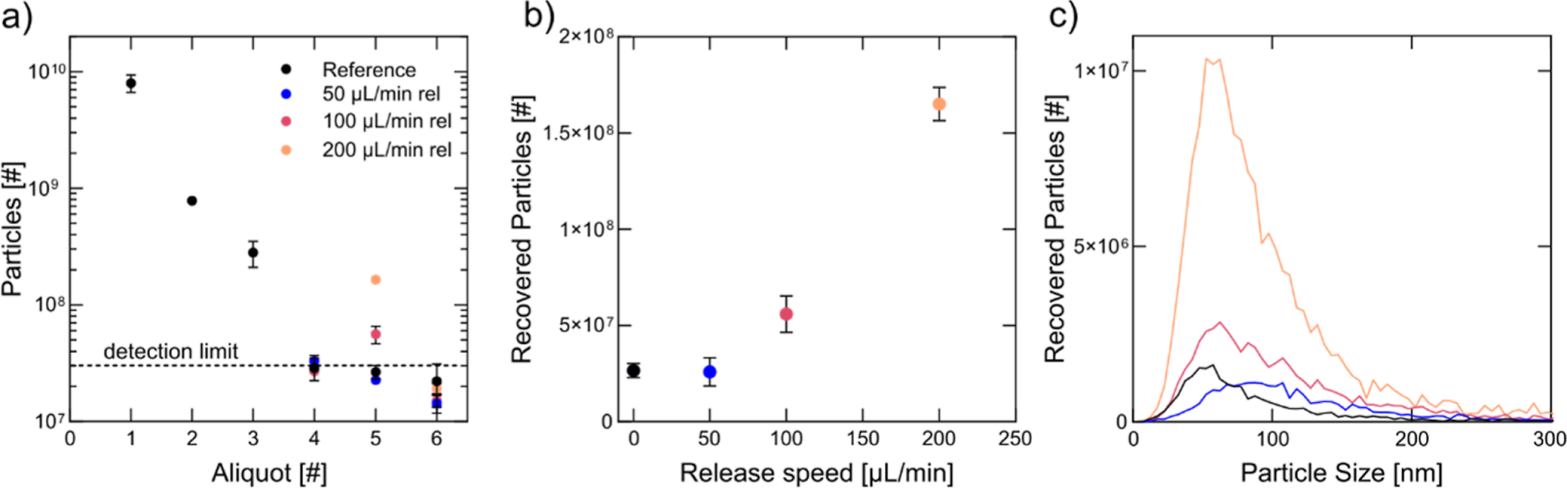
Release flow rate has a significant influence on retrieved particle number. (a) NTA measurement of the particle number in the collected 100 μL aliquots. The flow speed with which particles are released after turning off the ultrasound significantly influences the particle number in aliquot 5. (b) Released particle number in aliquot 5 for the different release flow speeds. The faster the release flow speed is chosen, the more particles can be released from the packed bed. (c) NTA measurement of particle size vs particle number in aliquot 5. Only the average is displayed for a clear representation. Plots containing error bars can be found in the Supporting Information (Figure S4). All experiments show the highest particle distribution in the small EV size range.

As outlined in the Experimental Procedure, adjustments to the release protocol significantly increased the number of particles in the fifth aliquot. Specifically, we observed that an abrupt increase in flow rate immediately after turning off the ultrasound enhanced particle recovery. To investigate this further, we varied the release flow rate. When maintaining the same flow rate during release as in the trapping phase (50 μL/min), no notable increase in recovered particle count was observed. However, a substantial rise in particle number occurred when the flow rate was abruptly increased (Figure 8a/b).

Regardless of the release flow rate, the recovered particles consistently fell within the size range of small EVs (Figure 8c). The observed increase in particle number may be attributed to reversibly bound particles, likely retained via hydrophobic/hydrophilic or electrostatic interactions. Particles brought into close proximity to the seed particles by acoustic forces might adhere weakly but require a strong hydrodynamic push for release. Experimental validation of this hypothesis is planned for a future study.

**Figure 9:**
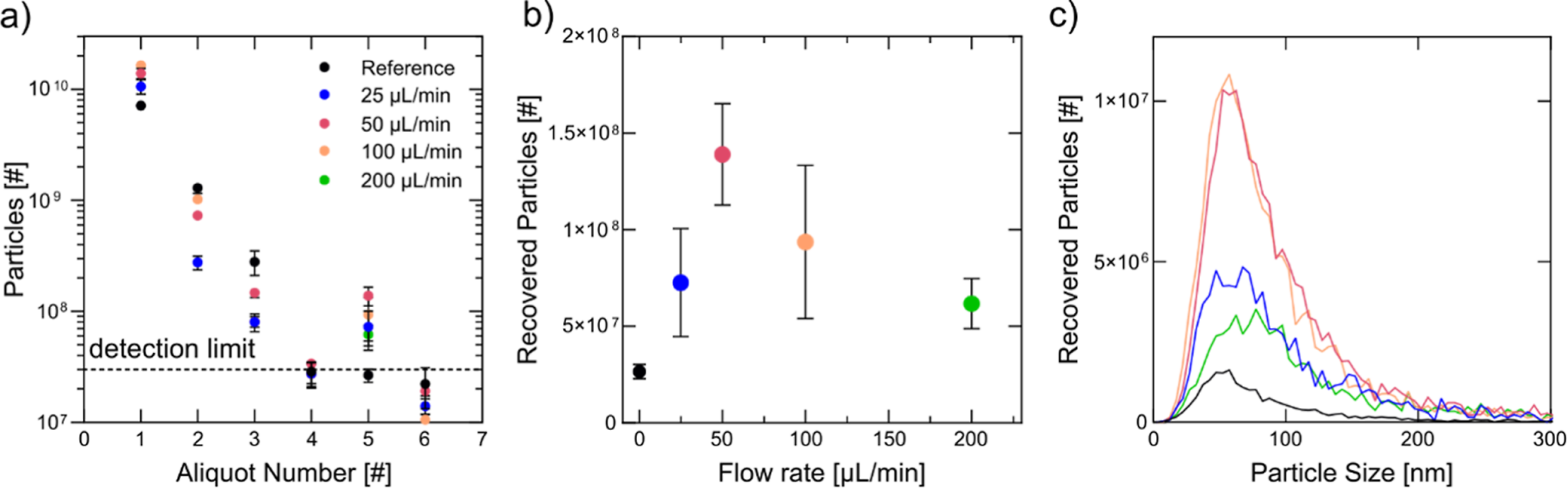
Flow rate influences the number of particles that can be trapped. (a) NTA measurement of the particle number in the collected 100 μL aliquots. The total flow speed with which particles flushed through the trapping device had a significant influence on number of particles in all aliquots. (b) Released particle number in aliquot 5 for the different total flow speeds. The optimal flow rate with which particles were flushed through the device was found to be 50 μL/min (c) NTA measurement of particle size vs particle number in aliquot 5. Only the average is displayed for a clear representation. Plots containing error bars can be found in the Supporting Information (Figure S-2). All experiment show the highest number of particles in the small EV size range.

Optimizing the release protocol by rapidly increasing the flow rate to 200 μL/min immediately after ceasing ultrasound, we then applied this method across experiments with varying total flow rates. The total flow rate influenced particle numbers in all aliquots. As seen in the PS particle experiments, a flow rate of 25 μL/min resulted in a significantly higher proportion of adsorbed particles (Figure 9a), which could not be efficiently released, even with the increased flow rate.

The highest recovery was observed at 50 μL/min (Figure 9b), consistent with previous experiments (Figure 4d). At flow rates exceeding 50 μL/min, stronger hydrodynamic forces likely displaced particles from the packed bed before effective trapping could occur. Regardless of the flow rate, the size distribution of released particles in the fifth aliquot consistently matched that of small EVs (Figure 9c).

In summary, our system efficiently traps, washes, and releases high numbers of EV-sized particles from just 4 μL of blood plasma. The released fraction contains less than 2 μg/mL of background protein, making it well-suited for downstream EV proteome analyses.

## Conclusion and Outlook

In this study, we developed and demonstrated a novel acoustofluidic system for the selective trapping and release of extracellular vesicles (EVs) from microliter-scale blood plasma samples. Our platform utilizes acoustic forces to efficiently capture EVs, which can then be released in a controlled manner by modulating flow dynamics. Notably, the system isolates 2 × 10^8^ EV-sized particles in 100 μL of eluate from just 4 μL of blood plasma, with minimal protein contamination (<2 μg/mL), making it highly suitable for downstream EV proteome analysis. The true particle concentration is likely even higher in a smaller subfraction of the eluate; however, current NTA technology limits our ability to analyze lower sample volumes.

Through frequency sweeping, we demonstrated robust particle trapping across a broad range (0.45–4 MHz). Additionally, we found that higher applied power, lower flow rates, and reduced inserted particles all contributed to enhanced recovery. We successfully demonstrated the trapping and release of 25 nm particles, with a trapping capacity of approximately 7 × 10^9^ particles, highlighting the system’s potential for nanoscale applications.

Despite these promising results, several aspects warrant further investigation. The observed decline in recovery at low flow rates (<50 μL/min) remains an open question, as does the potential capture of even smaller EV subpopulations that fall below the detection threshold of our current NTA measurements. Moreover, while our results confirm efficient EV trapping and release, further studies are needed to dissect the influence of hydrodynamic forces, hydrophobic/hydrophilic interactions, and electrostatic effects on particle retention and release, which could help refine system performance.

Looking ahead, future work will explore the impact of bead size and material composition on EV separation, with the goal of isolating distinct EV subpopulations. Given its ability to rapidly process small sample volumes with high purity, acoustofluidic chromatography holds significant potential for clinical translation, particularly in early disease detection and diagnostic applications.

## Supporting Information Available

Picture of the trapping device and packed bed (Figure S-1), photograph of the Experimental Setup (Figure S-2), plot of trapping experiments corresponding to Figure 4d (Figure S-3), NTA measurements including error bars from Figure 8c and Figure 9c (Figure S-4), Table with Performance metrics corresponding to Figure 4b (Table S-1), Figure 4c (Table S-2), Figure 4d (Table S-3), and Figure 5 (Table S-4) (PDF)
