# Supplementary material for: Acoustofluidic Chromatography for Extracellular Vesicle Enrichment from 4 μL Blood Plasma Samples
Source: Anal Chem. 2025 Mar 13;97(11):6049–58. doi: 10.1021/acs.analchem.4c06105 (PMC11948168; doi:10.1021/acs.analchem.4c06105)
Supplement: Supplementary file 1 — ac4c06105_si_001.pdf [file ac4c06105_si_001.pdf]

Supporting Information
Acoustofluidic Chromatography for Extracellular Vesicle Enrichment from 4 µL Blood Plasma Samples

Michael S. Gerlt[1] and Thomas Laurell[1,*]
[1]: Acoustofluidics Group, Department of Biomedical Engineering, Lund University, Sweden
*: E-Mail: thomas.laurell@bme.lth.se

Table of contents

1. Supporting Figures S3
A Figure S-1 …………………………………………………………………………………….. S3
B Figure S-2 …………………………………………………………………………………….. S3
C Figure S-3 …………………………………………………………………………………….. S4
D Figure S-4 …………………………………………………………………………………….. S4
2. Supporting Tables S5
A Table S-1 …………………………………………………………………………………….. S5
B Table S-2 …………………………………………………………………………………….. S5
C Table S-3 …………………………………………………………………………………….. S5
D Table S-4 …………………………………………………………………………………….. S6

Supporting Figures

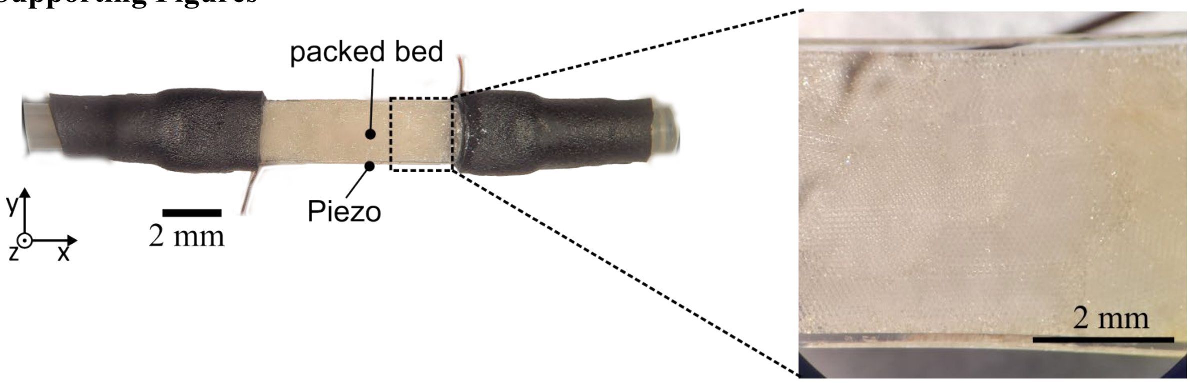

Figure S-1: Photograph of the trapping device taken from the top. Black heat shrink tubing and transparent silicone tubing can be seen at the capillary in- and outlet. Inset: Microscope picture of the packed bed showcasing the hexagonal patterns formed indicating good packing.

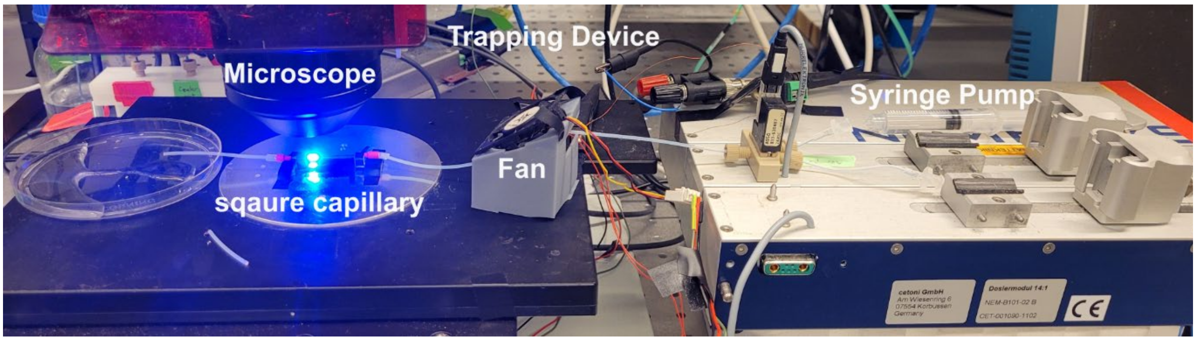

Figure S-2: Photograph of the Experimental Setup including Syringe Pumps, trapping device with fan on top and the analysis capillary with microscope for fluorescence analysis.

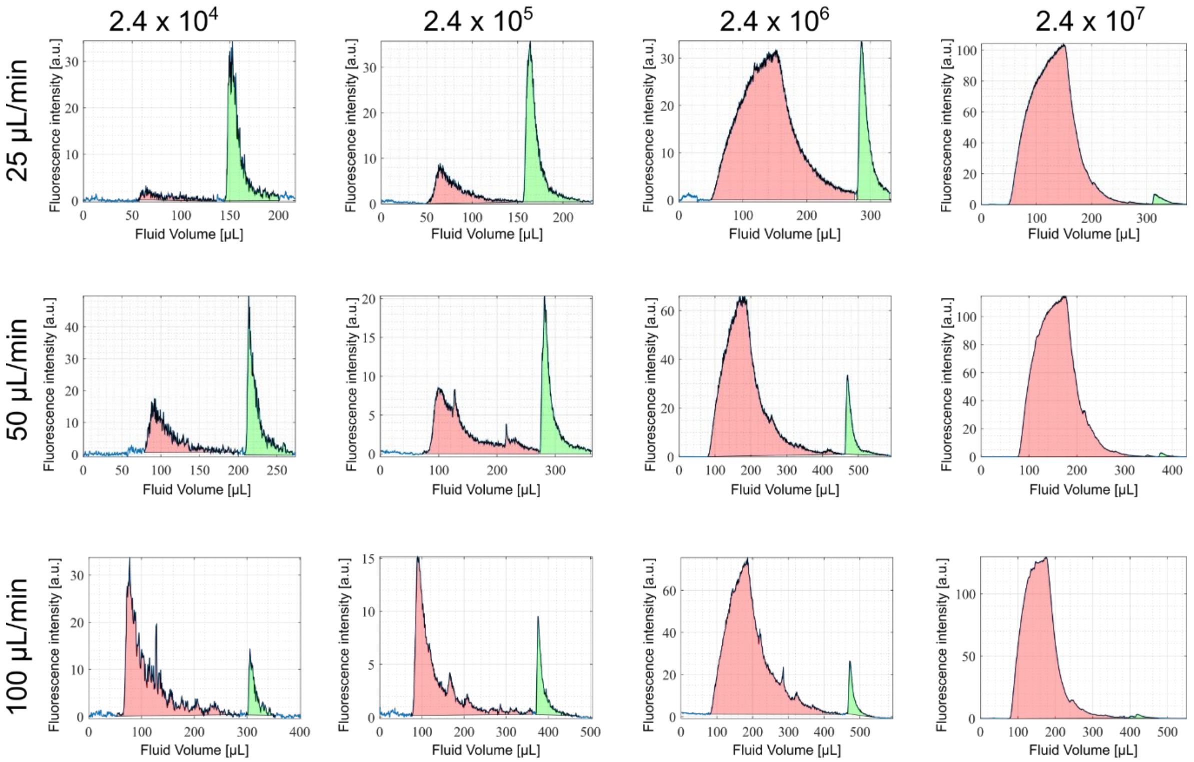

Figure S-3: Fluorescence intensity v.s. fluid volume for various flow rates and input particle amount corresponding to Figure 4d. The highest concentration of recovered particles is eluted in a volume of ~20 µL, independent of flow rate and input particle concentration.

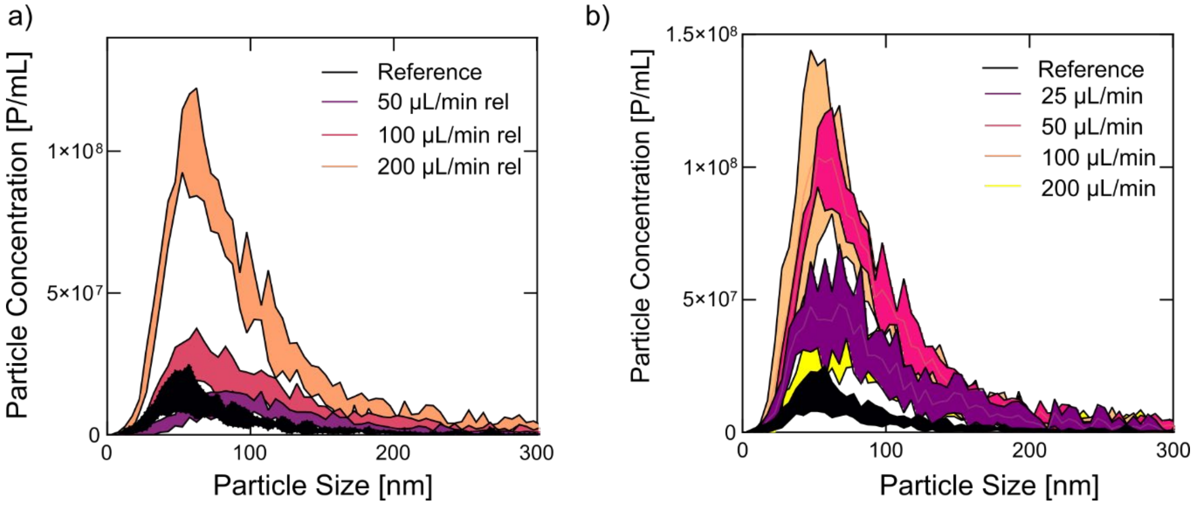

Figure S-4: NTA measurements including error bars from a) the experiment with varying release flow rate (Fig 8c) and b) the experiment with varying total flow rate (Fig 9c)

Supporting Tables

Table S-1: Performance metrics data corresponding to the frequency sweep experiment in figure 4b.
Particles: 2.46×10^6 2 µm PS particles were inserted. Power: 900 mW. Flow rate: 100 µL/min.

frequency [MHz]	0.45	1.19	1.60	2.10	2.62	3.30	4.00
Recovery [%]	4.5 ± 0.6	2.9 ± 1.2	3.2 ± 0.3	2.3 ± 0.3	1.9 ± 0.1	1.4 ± 0.1	0.6 ± 0.1
Flow Through [%]	78.0 ± 1.5	73.5 ± 8.5	72.3 ± 2.9	74.6 ± 4.1	78.1 ± 2.5	72.0 ± 2.5	84.3 ± 4.2
Adsorbed [%]	17.5 ± 1.2	23.6 ± 9.7	24.5 ± 2.6	23.1 ± 4.0	20.0 ± 2.4	26.5 ± 2.6	15.1 ± 4.3

Table S-2: Performance metrics data corresponding to the power sweep experiment in figure 4c.
inserted particles: 2.46×10^6 2 µm PS. Frequency: 450 kHz. Flow rate: 100 µL/min.

Power [mW]	55.00	110.00	225.00	450.00	900.00
Recovery [%]	0.8 ± 0.3	1.4 ± 0.4	2.1 ± 0.5	2.7 ± 0.5	4.5 ± 0.6
Flow Through [%]	82.6 ± 8.0	81.5 ± 5.3	83.4 ± 4.3	80.5 ± 3.0	78 ± 1.5
Adsorbed [%]	16.6 ± 8	17.2 ± 5.4	14.6 ± 3.9	16.8 ± 3.0	17.5 ± 1.2

Table S-3: Performance metrics data corresponding to 4d. See also Supp fig. S-3 for corresponding fluorescence graphs.
inserted particles: 2 µm PS particle. Frequency: 450 kHz.

Inserted Particle amount	2.40×10^4	2.40×10^5	2.40×10^6	2.40×10^7
**25 µL/min**				
Recovery [%]	30.0 ± 2.4	33.0 ± 2.0	5.1 ± 0.9	0.4 ± 0.1
Flow Through [%]	6.6 ± 1.5	11.3 ± 2.2	35.5 ± 0.4	25.5 ± 3.3
Adsorbed [%]	63.3 ± 1.0	55.7 ± 4.0	59.4 ± 1.2	74.1 ± 3.3
Recovered Particles [#]	$7.2 \times 10^3 \pm 5.7 \times 10^2$	$7.9 \times 10^4 \pm 4.8 \times 10^3$	$1.2 \times 10^5 \pm 2.1 \times 10^4$	$9.1 \times 10^4 \pm 1.9 \times 10^4$
**50 µL/min**				
Recovery [%]	35.4 ± 4.5	42.9 ± 3.2	7 ± 0.9	0.2 ± 0.1
Flow Through [%]	51.6 ± 10.8	52.6 ± 5.0	85.7 ± 7	47.2 ± 2.1
Adsorbed [%]	13.0 ± 6.3	4.5 ± 1.8	7.3 ± 6.8	52.4 ± 2.4
Recovered Particles [#]	$8.5 \times 10^3 \pm 1.1 \times 10^3$	$1.0 \times 10^4 \pm 7.7 \times 10^3$	$1.7 \times 10^5 \pm 2.2 \times 10^4$	$5.5 \times 10^4 \pm 1.6 \times 10^4$
**100 µL/min**				
Recovery [%]	11.4 ± 0.9	13.2 ± 2.1	4.5 ± 0.6	0.4 ± 0.05
Flow Through [%]	66.2 ± 6.1	62.2 ± 6.5	78.0 ± 1.5	51.4 ± 1.7
Adsorbed [%]	22.4 ± 6.3	24.7 ± 8.6	17.5 ± 1.2	48.2 ± 1.7
Recovered Particles [#]	$2.7 \times 10^3 \pm 2.2 \times 10^2$	$3.2 \times 10^4 \pm 5.0 \times 10^3$	$1.1 \times 10^5 \pm 1.4 \times 10^4$	$1.0 \times 10^5 \pm 1.2 \times 10^4$

Table S-4: Performance metrics data corresponding to figure 5.
inserted particles: 0.27 µm PS. Power: 450 mW. Frequency: 450 kHz. Flow rate: 50 µL/min.

Inserted Particle amount	9.70x104	9.70x105	9.70x106	9.70x107
Recovery [%]	7.8 ± 0.5	2.9 ± 1.0	0.4 ± 0.1	0.2 ± 0.03
Flow Through [%]	64.7 ± 1.7	57.8 ± 4.7	52.9 ± 1.1	46.7 ± 0.3
Adsorbed [%]	27.5 ± 2.0	39.4 ± 5.7	46.7 ± 1.0	53.1 ± 0.2
Recovered Particles [#]	$7.6x10^3 \pm 4.5x10^2$	$2.8x10^4 \pm 1.0x10^4$	$4.1x10^4 \pm 9.6x10^3$	$1.70x10^5 \pm 2.7x10^4$
